# The utility and cost of embedding geriatric expertise in a tertiary referral renal clinic

**DOI:** 10.1093/ckj/sfag132

**Published:** 2026-05-20

**Authors:** Virginia Aylett, Samuel D Relton, Zoe Rogers, Dan Howdon, Anna Winterbottom, Andrew Mooney

**Affiliations:** Department of Healthcare for the Elderly, Leeds Teaching Hospitals NHS Trust, Leeds, UK; Leeds Institute of Health Sciences, School of Medicine, University of Leeds, Leeds, UK; Leeds Institute of Health Sciences, School of Medicine, University of Leeds, Leeds, UK; Leeds Institute of Health Sciences, School of Medicine, University of Leeds, Leeds, UK; Department of Healthcare for the Elderly, Leeds Teaching Hospitals NHS Trust, Leeds, UK; Leeds Institute of Health Sciences, School of Medicine, University of Leeds, Leeds, UK; Department of Healthcare for the Elderly, Leeds Teaching Hospitals NHS Trust, Leeds, UK; Leeds Institute of Health Sciences, School of Medicine, University of Leeds, Leeds, UK

**Keywords:** conservative management, decision-making, dialysis, frailty, geriatric

## Abstract

**Background:**

To address the changing demographic and increasing frailty and comorbidity of people referred to renal services, we initiated novel, routine, embedded, consultant-led, focused geriatric assessment of a selected group of patients in our Renal Low Clearance Clinic, seeking effects on treatment decision-making, patient outcomes and undertaking a health economic analysis.

**Methods:**

A total of 133 patients fulfilling study-developed referral criteria received focused geriatric assessment. Short-term results (treatment decisions) of all 133 patients, plus long-term (survival) data for the first 77 patients for whom we have 3 years’ follow-up are presented. Health economic analysis compared the cost of employing the Geriatrician versus avoiding unnecessary/futile dialysis access (arteriovenous fistula) creation based on historic rates in our own unit.

**Results:**

Starting in 2018, 77 patients were reviewed before suspension enforced by the COVID-19 pandemic in March 2020, and a further 56 since resumption between July 2021 and January 2023 [mean age 78 (range 62–92) years; 70% male]. Following focused geriatric assessment, the number of patients undecided about treatment changed from 43 to 3; those choosing dialysis reduced from 80 to 44 and those choosing conservative management (CM) increased from 10 to 74. The number of advance care plans made increased from 0 to 77, and recorded resuscitation decisions from 6 to 42. Thirty-six months after focused geriatric assessment, the survival rate in the group choosing dialysis was 50% and in the CM group was 33%; most deaths were unrelated to renal failure and there was a trend towards clinical frailty scores impacting outcome more than treatment choice. Health economic analysis demonstrated that the costs of providing this review were more than offset by reductions in unnecessary/futile fistula formation.

**Conclusions:**

Routine, protocol-supported focused geriatric assessment in a tertiary referral renal service appears cost-effective and is associated with improved dialysis decision-making, advance care-planning and resuscitation decision-making.

KEY LEARNING POINTS **What was known:**

- There are increasing numbers of patients who are elderly, frail or comorbid reaching end-stage kidney disease.
- Geriatric assessment has been proposed to support frailty assessment and treatment (dialysis) decision-making in this group, but the cost of implementing this is frequently cited as a barrier.

**This study adds:**

- Not only confirmation that focused geriatric assessment is clinically useful for identifying frailty syndromes in advanced kidney disease and supporting treatment decisions, but also that this intervention appears to be cost-effective by reducing futile fistula formation surgery alone.

**Potential impact:**

- The demonstration that focused geriatric assessment of selected elderly people with chronic kidney disease is a likely cost-effective intervention which enables identification of geriatric syndromes and prevention of futile interventions will enable other units to confidently introduce this innovation, and/or acquire the resources to do so.

## INTRODUCTION

The Renal Low Clearance Clinic (LCC) in Leeds is a tertiary referral centre providing a service to a population of around 1.9 million people in West Yorkshire in the north of England, UK. It was created in 2001 to review patients with declining renal function and prepare them for dialysis and transplant [renal replacement therapy (RRT)]. Referral into the service was protocolized at estimated glomerular filtration rate (eGFR) of 18–20 mL/min/1.73 m^2^; patients are counselled to reach a preferred RRT decision which is recorded electronically.

Following the publication of the Renal National Service Framework in 2005 [1], National Institute for Health and Care Excellence (NICE) Clinical Guideline 39 [2] recommending anaemia treatment in non-dialysis patients and the Renal Quality Outcome Framework rewarding primary care identification of chronic kidney disease (CKD) [3], the size of the service had increased 3-fold and comprised an increasingly elderly population with significant comorbidity, many of whom would not have been previously considered suitable for RRT. Therefore, in 2008 a Palliative Care Consultant was embedded into the service, offering conservative management (CM) (i.e. non-dialysis) as an alternative to RRT. We reported the results of the first 4 years’ experience of this clinic in 2013 [4]; similar results were subsequently reported from other units [5]. Currently, around 25% of all patients and 40% of those aged over 70 years under review in the LCC are under the care of this CM service.

However, our original report of outcomes in patients in the clinic aged over 70 years showed that even among the cohort that chose dialysis, 25% died before starting dialysis, and 30% were alive but had not started dialysis 4 years after making their original decision [4]. Thus, the LCC accumulated increasing numbers of people who may have been suitable for RRT at the time of referral to the clinic and when they made their modality choice, but some years later, having not yet required dialysis, might no longer benefit from this treatment.

Dialysis decision-making is difficult in such patient groups owing to the uncertainty of the prognosis and benefit of dialysis in the face of multiple comorbidities; these challenges have been summarized and been the subject of guidance from the European Renal Best Practice Group [6]. This was additionally challenging in our cohort who had already undergone an earlier previous process of pre-dialysis education to facilitate treatment decision-making. Furthermore, owing to the uncertainty around benefits of RRT, the numbers of patients prevalent in the Leeds LCC who remained undecided on preferred RRT modality even after counselling in the service rose from 0% in 2010 to 11% in 2018, and over 95% of those ‘undecided’ were aged over 70 years.

Geriatric review, bringing expertise in frailty and comorbidity, has been shown to be valuable in treatment decision-making in a number of other services; this may be a model comprising full comprehensive geriatric assessment, utilizing the skills of the full multidisciplinary team [7, 8], or may be limited to Geriatrician input that focuses on specific problems such as post-operative surgical care [9].

We speculated whether similar expertise could be valuable in this service. A Geriatrician was therefore recruited into the Leeds LCC service to provide a holistic overview that sought to identify geriatric syndromes, assess the impact of comorbidities on patients’ lives, and use this information to explore treatment decisions as well as discuss resuscitation and advance care planning.

We labelled this review a ‘focused geriatric assessment’, rather than comprehensive geriatric assessment, as it focused on outcomes important and relevant to the renal service in which it took place, but did not include the routine involvement of other multidisciplinary team professionals.

## MATERIALS AND METHODS

A Geriatrician was embedded into the LCC in July 2018 on an alternate-weekly basis to see three new and one to two follow-up patients per clinic. Originally, there were three main aims for the new service:

- identification of co-existent geriatric syndromes, including falls, frailty and cognitive impairment; every patient was assessed for and given a clinical frailty score (CFS) [10];
- holistic overview and exploration of the patient’s treatment goals, including consideration of other life-limiting conditions as well as the patient’s wishes and understanding of their illness, and an assessment of how these aligned with current treatment plans; and
- undertake resuscitation and advance care planning discussions.

At its inception, the plan was to arrange focused geriatric assessment of any patient aged over 75 years who was deemed to be unsuitable for a kidney transplant. These patients were identified by the pre-dialysis nurses, who undertake home visits for all patients considering RRT, and initially included some patients who had already selected CM of their advanced CKD.

Two months after the clinic started, following the first PDSA (Plan, Do, Study, Act) cycle [11], patients who had already chosen CM prior to geriatric review were no longer referred to this service as it was felt that they derived little benefit. Through further PDSA cycles the pre-dialysis nurses became empowered to refer patients who fell outside the initial referral criteria; thereafter, through a ‘tally chart’ exercise [12] with support from the Specialised Clinical Frailty Network [13], we were able to identify a number of reasons to trigger geriatric review and these remain our referral criteria (Table 1).

**Table 1:** Indicators in the renal clinic to trigger geriatric review.

**Themes in identifying patients for focused geriatric assessment**
1. Recognition of frailty phenotypes using the CFS
2. Identification of falls, cognitive impairment and other frailty syndromes
3. Discovery of unexpected home circumstances during home visits
4. Patients finding it difficult to come to a decision regarding RRT
5. Patients or their families appearing to have unrealistic expectations of treatment outcomes
6. Patients aged >80 years with a plan to start dialysis

We measured treatment decision before and after geriatric review, CFS [10] at time of geriatric review, resuscitation decisions (on ‘ReSPECT’ forms [14]—an electronic resource to record resuscitation and advance care plans which in Leeds can be viewed by both primary- and secondary-care providers irrespective of where the form is originally completed) and survival (mean and standard deviation) from the date of geriatric review. We also studied the health economic impact of the intervention (see below). Data were collected contemporaneously and outcomes for every patient followed up through the focused geriatric assessment service were recorded.

### Health economic evaluation

We wanted to undertake a cost-effectiveness analysis of the geriatric review of our patients. As we did not collect quality of life data, a full analysis was not possible, and we chose instead to perform an exploratory cost-effectiveness analysis, assuming at minimum no harm from the new intervention and measuring the cost only in terms of reduction of ‘useless’ fistula creation for patients.

**Figure 1:**
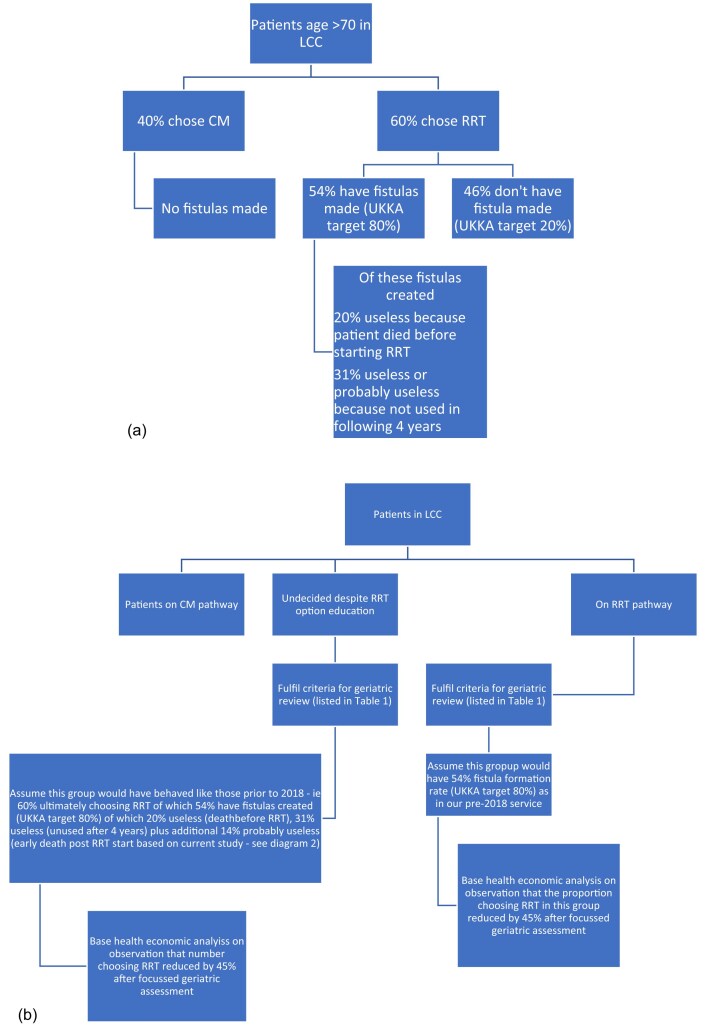
(**a**) Leeds LCC service prior to introduction of geriatric review (pre-2018) [4]. (**b**) Leeds LCC service after introduction of geriatric review (post-2018).

To do this, we used our historical published data on the number of patients who chose dialysis after establishment of our CM service, but prior to the introduction of geriatric review [4], our rates of fistula formation among haemodialysis patients in the relevant period in the UK Renal Registry [15] [plus an analysis using historical UK Kidney Association (UKKA) recommended rates of fistula formation [16]] and the survival rates to dialysis and on dialysis among this group based on our historical data, to determine how many of the undecided group would have been likely to choose RRT without the newly introduced geriatric review. This process of comparison of the novel geriatric review with historical data and the assumptions we made are summarized in Fig. 1a and b.

We considered the budgetary impact solely in terms of the avoidance of ‘useless’ fistula creation and defined this as occurring for two reasons:

- definitely useless, where the patient either dies before RRT takes place, or had not started RRT within 3 years;
- definitely or probably useless, as (i), additionally including patients who received RRT for < 3 months before dying.

We estimate the number in each as follows:


\begin{eqnarray*}
{\mathrm{Useless\ fistulas\ avoided}} = {\mathrm{Avoided\ RRT}} \times {P}_{{\mathrm{fistula}}} \times {P}_{{\mathrm{useless}}}
\end{eqnarray*}

where Avoided RRT is the number of patients estimated to opt for CM rather than RRT as a result of their attendance at the clinic, defined below; *P*_fistula_ is the proportion of patients who opt for RRT for which fistulas are in practice created [this is estimated for the actual rate for Leeds according to the 2018 UK Renal Registry Report (54%) and the target rate set by the UK Kidney Association (80%); 2018 was the year the service was initiated]; and *P*_useless_ is the proportion of fistulas created that are estimated to be useless (according to the two earlier definitions), in our follow-up sample of 28 patients.

The number of patients estimated to opt for CM rather than RRT as a result of clinic attendance is given by:


\begin{eqnarray*}
{\mathrm{Avoided\ RRT}} &=& \left( {{\mathrm{RR}}{{\mathrm{T}}}_{{\mathrm{before}}} - {\mathrm{RR}}{{\mathrm{T}}}_{{\mathrm{after}}}} \right) \\&&+ \left( {{\mathrm{Undecide}}{{\mathrm{d}}}_{{\mathrm{before}}} \times {P}_{{\mathrm{RRT}},{\mathrm{\ no\ clinic}}}} \right)\\&&- \left( {{\mathrm{Undecide}}{{\mathrm{d}}}_{{\mathrm{before}}} \times {P}_{{\mathrm{RRT}},{\mathrm{\ clinic}}}} \right)
\end{eqnarray*}

where RRT_before_ is estimated by the number of patients planning to choose RRT over CM before clinic attendance in our main sample of 133 patients; RRT_after_ is estimated by the number of patients planning to choose RRT over CM after clinic attendance in our main sample of 133 patients; Undecided_before_ is estimated by the number of patients who are undecided before clinic attendance in our main sample of 133 patients; *P*_RRT_, no clinic is estimated by the proportion of previously undecided patients who would choose RRT in the absence of the clinic, based on historical data from 441 patients [4]; and *P*_RRT,clinic_ is estimated by the proportion of previously undecided patients who chose RRT following clinic attendance in our main sample of 133 patients.

This implies that there are two groups of patients who contribute to Avoided RRT: patients who post-clinic opted for CM who were either (i) planning to choose RRT or (ii) undecided and would be expected to choose RRT, but who post-clinic chose CM. The figures for each of these are taken from the most relevant available data, and sampling uncertainty around them characterized through bootstrap resampling.

After estimating the number of useless fistulas avoided, we multiply this value by the estimated cost of fistula creation. This cost was taken from 2021–22 National Cost Collection data (https://www.england.nhs.uk/costing-in-the-nhs/national-cost-collection/) and estimated at £3109.90, corresponding to the cost of a day case for ‘Open Arteriovenous Fistula, Graft or Shunt Procedures’.

We assumed that the cost of provision of the clinic can be estimated solely by the full absorption cost of the Geriatrician’s time. The Personal Social Services Research Unit’s 2021/22 estimates [17] are that 1 h of a consultant’s time is associated with a full cost (i.e. including salary, other benefits, qualification costs and overheads) of £145.

## RESULTS

The clinic started in July 2018, and ran until March 2020 when it was paused due to the COVID-19 pandemic. It resumed in July 2021 and continues in the same format.

Up until January 2023, a total of 133 patients were seen, with 77 seen up to March 2020, and a further 56 since then. All appointments were face to face, and the majority of patients were accompanied by partners or family members (120/133, 90%). Approximately one-third of patients (39) had one or more follow-up visits, the remainder being seen just once. Seventy percent of patients were male, with a mean age of 78 (range 62–92) years.

### Short-term outcomes

Short-term outcomes are reported for all 133 patients reviewed up to January 2023.

#### Identification of co-existent geriatric syndromes

All patients underwent frailty scoring on the CFS [10] and 54.5% of patients were at least mildly frail, with a score of 4 or more. Among the 133 focused geriatric assessments, 9 patients were found to have previously unidentified continence problems, 7 patients were identified to be having falls and were referred on to specialized services, and an additional 2 patients were given exercise advice in the consultation to improve strength of relevant muscle groups. Additionally, 19 patients were found to be living with cognitive impairment; in 2 cases this was sufficiently severe to be influential in their ability to make a decision about treatment options. Separately, 2 of the 19 patients reported that they did not wish to have onward referral (see below) from the Geriatrician for further assessment of their cognitive impairment; additionally, 1 patient previously thought to have cognitive impairment due to vagueness and repetitiveness was instead diagnosed with complex partial seizures. Due to the tertiary nature of the clinical service, direct onward referral to specialized services for these geriatric syndromes was only possible for the 50% of patients who lived within the Leeds secondary care catchment area; for the remainder (those whose secondary care was delivered by other regional hospitals outside Leeds) we made recommendations to primary care physicians.

#### Treatment goals

Forty-six percent of patients who came to the clinic subsequently agreed on a different management plan. The breakdown can be seen in Table 2.

**Table 2:** Treatment decisions and plans before and after geriatric review.

Treatment decision	Before clinic (no. patients)	After clinic (no. patients)
Conservative management	10	74 (includes 6 discharged to GP)
RRT (haemodialysis or peritoneal dialysis)	80	44
Undecided	43	3^a^
Decision deferred		8^b^
Died before next Geriatrician follow-up appointment		4
Total	133	133

^a^Patients who remained unable to or refused to discuss treatment options.

^b^Patients reported would make decision closer to need for RRT but judged at focused geriatric assessment to be unlikely to reach this point. GP, general practitioner.

CM patients were only seen in the first 2 months after establishing the clinic whereafter they went straight to the CM clinic without additional geriatric review. A number of patients with a higher eGFR (e.g. 18 mL/min/1.73 m^2^) or very slowly declining renal function failed to make a decision regarding RRT, despite extensive discussions, in which case their treatment decision was deferred. None of these patients subsequently went on to receive RRT. Four patients died between their first and follow-up appointment with the clinic (none received RRT). Of those patients who agreed a different management plan following the consultation, the majority opted for CM. However, one patient switched from having chosen CM prior to geriatric review to RRT, and three who opted for CM following geriatric review later changed back to RRT.

#### Resuscitation and advance care plan discussions

Prior to the clinic visit, only six patients already had a cardio-pulmonary resuscitation (CPR) decision recorded.

A further 43 patients (32%) formally completed CPR decisions (the vast majority ‘Do Not Attempt CPR’) following the appointment, and 87 patients (65%) had ‘ReSPECT’ forms completed [14] (i.e. resuscitation decision recorded and/or advance care plan made—see Materials and methods).

### Longer-term outcomes

Longer term outcome data were collected for all 77 patients who were seen prior to suspension of the clinic due to the Covid-19 pandemic in March 2020, with follow-up ranging from 2–4 years.

#### Patients choosing RRT

**Figure 2:**
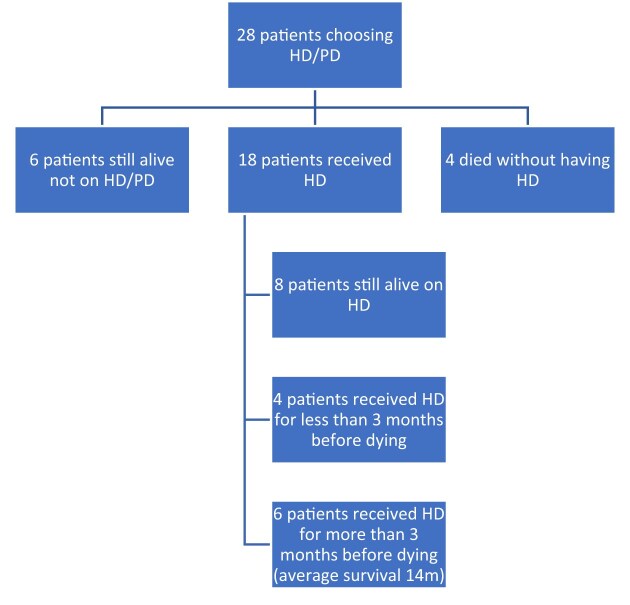
Consort diagram of patients reviewed by Geriatrician and still choosing RRT after 3 years of follow-up.

28 of these 77 patients chose RRT (27 HD and 1 PD). The outcome for these patients is shown in Fig. 2.

The survival rate for this group of patients was 50% at 3 years. Of those who died without receiving dialysis, two died following myocardial infarction (one in cardiogenic shock), one died of biventricular heart failure and one of pneumonia and critical aortic stenosis. Of those who died after receiving dialysis, three died from chest infection (including one COVID-19 infection) and another from chronic obstructive pulmonary disease without infection; two died from sudden cardiac death; two following elective dialysis withdrawal; one from alcoholic liver disease; and one in multi-organ failure from sepsis of unknown cause for which dialysis was initiated as part of their treatment during their terminal illness.

#### Patients choosing CM

**Figure 3:**
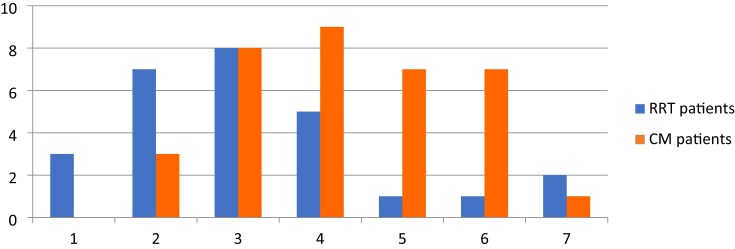
CFS vs treatment choice after Geriatrician review.

The frailty scores were generally higher for this group (Fig. 3), containing a larger number of patients with CFS 5–7 than among those choosing RRT, though it can be seen that some frail patients chose RRT and some non-frail patients chose CM.

**Figure 4:**
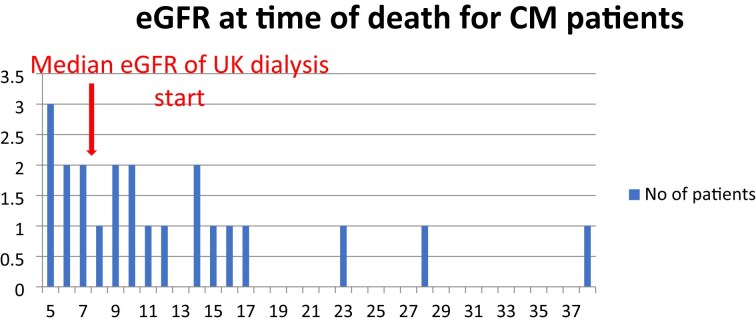
eGFR (mL/min/1.73 m^2^) at time of death for those choosing CM following Geriatrician review.

The survival rate for this group was lower than the RRT group at 33% at 3 years. Most patients choosing CM died with an eGFR significantly higher than the median level at which patients in the UK commence dialysis (Fig. 4) [15].

#### Comparison between frailty and treatment groups

**Figure 5:**
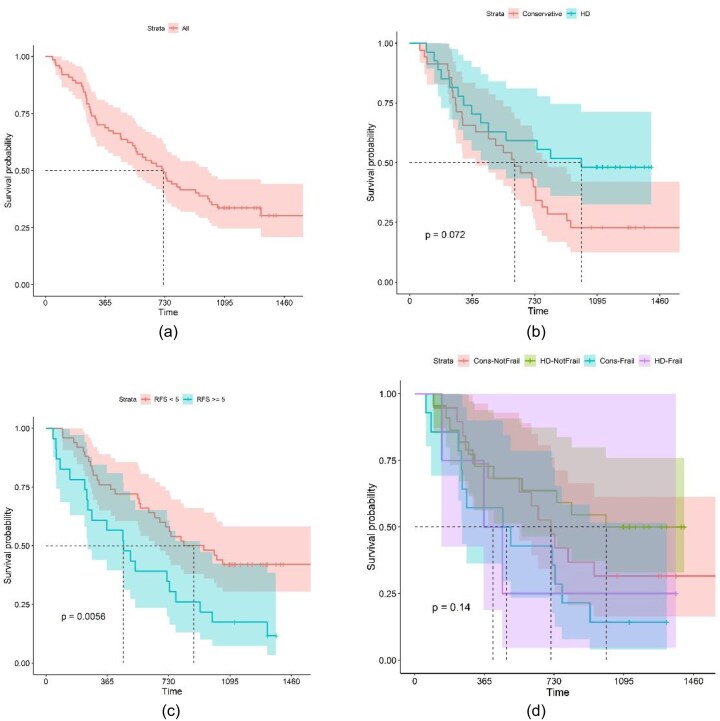
(**a**) Kaplan–Meier survival curve for all patients from date of Geriatrician review. (**b**) Kaplan–Meier survival curve from date of Geriatrician review divided by CM (red) and haemodialysis (HD) (green). (**c**) Kaplan–Meier survival curve from date of Geriatrician review divided by low and high frailty by CFS (CFS 1–4, red; CFS 5–7, green). (**d**) Kaplan–Meier survival curve from date of Geriatrician review divided by frailty and treatment choice [low frailty (CFS 1–4) HD, green; low frailty (CFS 1–4) CM, red; high frailty (CFS 5–7) HD, lilac; high frailty (CFS 5–7) CM, blue].

The Kaplan–Meier survival curves (with standard error in shaded area) for patients who underwent focused geriatric assessment is shown in Fig. 5, below. Median survival in the whole group from the date of geriatric review was about 2 years.

We measured survival having divided the groups by treatment choice following geriatric review and found that those choosing RRT tended to have longer survival than those choosing CM [833 (±492) days for those choosing dialysis and 677 (±443) days for those choosing CM irrespective of frailty status], but this did not achieve statistical significance (*P* = .072).

However, when the group was divided by CFS, there was a statistically significant difference in survival when those with a CFS of 1–4 were compared with those with a CFS of 5–7 [CFS 1–4 mean survival was 820 (±462) days and for CFS 5–7 was 577 (±447) days irrespective of choosing RRT or CM, *P* = .0056].

When CFS and treatment choice were combined and survival analysed, the numbers of patients in each group became too small to achieve statistical significance, but it was noteworthy that the more frail groups (CFS 5–7) had lower survival rates than the less frail groups (CFS 1–4) whatever treatment (RRT or CM) was chosen (i.e. frail patients choosing RRT experienced lower survival rates than less frail patients choosing CM).

### Health economic evaluation

Our health economic evaluation was based on comparing the cost of employing the Geriatrician in the renal service to the savings which might be accrued by avoiding creating fistulas in readiness for dialysis which were likely or definitely useless. To compare the likely fistula formation rate without the geriatric review, we used our published historic data [4] describing the number of patients choosing dialysis and the number in whom fistulas were made (and an additional analysis based on the number of fistulas recommended in incident haemodialysis patients at the time by the UKKA). Using these assumptions, Table 3a gives our estimates in total for the 133 patients seen by the clinic for a fistula rate of 54% (actual) and Table 3b for a fistula rate of 80% (UKKA-recommended target).

**Table 3a:** Estimated impact for 133 patients, based on 54% (actual 2018) fistula creation rate.

	Definitely useless	Definitely or probably useless
Fistulas averted, point estimate	12.07	16.89
95% CI (lower bound)	6.00	10.80
95% CI (upper bound)	18.00	22.80
Cost savings, point estimate	£37 550.92	£52 534.19
95% CI (lower bound)	£18 661.44	£33 590.59
95% CI (upper bound)	£55 984.32	£70 913.47

CI, confidence interval.

**Table 3b:** Estimated impact for 133 patients, 80% target fistula creation rate (UK Renal Registry target).

	Definitely useless	Definitely or probably useless
Fistulas averted, point estimate	17.89	25.03
95% CI (lower bound)	*8.89*	*16.00*
95% CI (upper bound)	*26.67*	*33.78*
Cost savings, point estimate	£55 631.00	£77 828.43
95% CI (lower bound)	*£27 646.58*	*£49 763.84*
95% CI (upper bound)	*£82 939.73*	*£105 057.00*

CI, confidence interval.

A similar analysis was performed on a per-person cost, dividing the figures by 133 to give the estimates in Tables 4a and 4b.

**Tables 4a:** Estimated impact per patient seen, 54% (actual 2018) fistula creation rate (the same estimates on a per-patient level, diving the numbers in Table 3 by 133).

	Definitely useless	Definitely or probably useless
Fistulas averted, point estimate	0.09	0.13
95% CI (lower bound)	*0.05*	*0.08*
95% CI (upper bound)	*0.14*	*0.17*
Cost savings, point estimate	£282.34	£394.99
95% CI (lower bound)	*£140.31*	*£252.56*
95% CI (upper bound)	*£420.93*	*£533.18*

CI, confidence interval.

**Table 4b:** Estimated impact per patient seen, 80% (target) fistula creation rate (the same estimates on a per-patient level, diving the numbers in Table 3 by 133).

	Definitely useless	Definitely or probably useless
Fistulas averted, point estimate	0.13	0.19
95% CI (lower bound)	*0.07*	*0.12*
95% CI (upper bound)	*0.20*	*0.25*
Cost savings, point estimate	£418.28	£585.18
95% CI (lower bound)	*£207.87*	*£374.16*
95% CI (upper bound)	*£623.61*	*£789.90*

CI, confidence interval.

Each clinic run by the Geriatrician lasted 4 h and involved meeting with three or four patients, after accounting for non-attenders. By assuming the lower end of number of patients seen, and that the Geriatrician was not able to undertake other relevant work during this 4-h period, we are able to estimate a plausible upper bound of £193 (145*4/3) for the cost of one single patient consultation. Comparing this cost with that saved in fistula costs per patient seen (£420–533 @ 54% fistula rate; £624–790 @ 80% fistula rate) strongly suggests that the operation of this clinic was substantially cost-saving. It is important to note that these costs are averaged per patient, and savings were made overall and irrespective of whether the individual patient chose CM or RRT.

## DISCUSSION

We believe there are three striking conclusions from this work. Firstly, that a Geriatrician is a useful resource in a renal clinic such as this; secondly, in this group of people frailty is highly impactful in predicting outcomes (emphasizing the value of utilizing frailty-assessment expertise); and finally that health economic analysis shows that this additional focused geriatric assessment is highly likely to be cost-effective by reducing futile dialysis fistula creation alone.

Although the management of advanced renal failure has evolved to include utilization of palliative care for elderly/frail patients and the recent recommendation of such treatment pathways in international guidelines [6], it has been repeatedly demonstrated that such care is more effectively and more frequently utilized by patients who have chosen CM rather than continuing to pursue dialysis treatment [4, 18–21], which in turn is contingent on the patient choosing this treatment pathway option.

Prior to geriatric review, 60% of the cohort described had chosen to pursue RRT and the remainder were still considering it. Most strikingly following geriatric review those who were undecided about treatment options reduced significantly, with an increased uptake of CM, a pattern seen repeatedly following geriatric assessment in other specialties [22]. In total, 46% of patients changed a treatment plan following the focused geriatric assessment which comprised a multidimensional holistic assessment administered by a Geriatrician alone (not a multidisciplinary comprehensive geriatric assessment), focused on the requirements of the renal clinic, but with the option to refer on to specialist therapy services where deemed appropriate.

We believe the patients’ outcomes following additional consideration of their original treatment choice triggered by the focused geriatric assessment merit further consideration. Many died before reaching dialysis and several (7/28 or 14% of those on the RRT pathway) within 3 months of starting (two after fewer than three sessions). Early dialysis death may well be under-reported in registry data [23]; we believe more frail patients would have received ‘futile’ dialysis had they remained on the RRT pathway.

CPR and advance care planning were increased following focused geriatric assessment, and very few had had discussions on this topic during routine renal review despite their age, frailty and comorbidity; a pattern seen in other disciplines [24]. We speculate that these discussions might facilitate future advance care planning among those that continue to opt for RRT, as studies show this group frequently wish to review their treatment decision later [25].

In our group, survival differences between those choosing RRT and CM are small, and it is noteworthy that frailty status correlated significantly with survival (irrespective of treatment choice), whereas treatment choice did not, suggesting that frailty might be more influential on outcome than planning for dialysis. Other studies at earlier stages of CKD have also shown that frailty is a more likely indicator of prognosis than stage of CKD itself [26]. Thus, assessment of frailty appears important in this group, but has been shown to be underestimated by nephrologists [27], emphasizing the value of the focused geriatric assessment. However, as shown in Fig. 3, even some non-frail patients choose CM over RRT, so principles of shared decision-making remain important in this patient group [28].

The focused geriatric assessment in our service was highly valued clinically but additionally in our health economic analysis, even with conservative estimates, it seemed likely that the cost of providing the assessment is more than offset by the savings made by not creating dialysis access where this is likely to be futile. There are likely additional cost reductions as multiple studies have shown reduced hospital admissions among patients not selecting dialysis treatment compared with those choosing CM [4, 18, 19, 29], and dialysis initiation is recognized to be frequently associated with physical [30, 31] and cognitive decline [32, 33]. It is also noteworthy that this service was provided to a catchment area of 1.9 million by a consultant Geriatrician attending for a half day every 2 weeks, and therefore extrapolating to the whole UK population of 70 million, this service could be provided to the entire population by fewer than two whole time-equivalent consultant Geriatricians.

However, more important than the healthcare costs, futile dialysis represents a potentially traumatic way of spending the last few weeks or months of life, with reduced likelihood of receiving palliative care review or social service support [4, 18, 19].

We acknowledge that the study is limited to a single centre, that the health economic evaluation looked only at the costs of fistula formation and no other healthcare costs (nor costs to the patient), and that the way we analysed our outcomes was reliant on our historical data (although these match those reported by others [5]) without an up-to-date comparator group; it is therefore possible that changes in dialysis preferences or advance care planning may have happened without geriatric review.

However, in summary, there is limited evidence that frail older people benefit from dialysis and employing a Geriatrician in a low clearance clinic may help patients come to a treatment decision that recognizes this fact, with patient, service and potential health economic benefits.

## DATA AVAILABILITY STATEMENT

The data that support the findings of this study are available from the corresponding author upon reasonable request.
